# Analyzing the influence of IL18 in regulation of YAP1 in breast oncogenesis using cBioportal

**DOI:** 10.1002/cnr2.1484

**Published:** 2021-06-30

**Authors:** Ayesha Rahman, Lingadahalli S. Shashidhara

**Affiliations:** ^1^ Department of Biology Indian Institute of Science Education and Research Pune Maharashtra India; ^2^ Department of Biology Ashoka University Sonipat India

**Keywords:** breast cancer, cBioPortal, IL18, YAP1

## Abstract

**Background:**

Yes‐associated protein 1 (YAP1) is responsible for tumor growth, progression and metastasis. The mechanisms controlling the generation and relative ratio of the functional YAP1 and other co‐factors are not well‐understood. Various literature reported that co‐factors like cytokines significantly influence signaling pathways to introduce epithelial immunity and regeneration, which later helps increase cancer‐related phenotypes. Among various cytokines, IL‐18 has emerged as a major player in inflammation and progression of different types of cancers. Till now, much information has not been known about the role of YAP1 in tumor aggressiveness and immune evasion in breast cancer with respect to IL‐18.

**Aim:**

We aimed to explore the effect of YAP1 in tumor aggressiveness and immune evasion in breast invasive carcinoma and metastatic breast cancer in the context of Interleukin‐18 (IL‐18) *in silico*.

**Methods and Results:**

We used publicly available data generated by The Cancer Genome Atlas (TCGA) Research Network through cBioportal web platform. Kaplan–Meier method was used to determine the overall survival and comparison between curves were made using Log‐Rank test. The *p* values were determined by Fisher's exact test with the null hypothesis. Correlation plots were analyzed by comparison with gene copy numbers from the GISTIC2.0, available through cBioportal.

Our analyses suggest that IL‐18 influences YAP1 expression in breast oncogenesis via Interferon‐gamma (IFN‐γ) production. Patients having a higher expression of IL‐18 possess a better prognosis and higher YAP1 expression with lower IL18 drives to poor clinical results in breast cancer.

**Conclusion:**

This can provide new approaches to better understand the relation between YAP1 and IL‐18 in breast cancer progression by performing in vitro and in vivo studies. Also, IL‐18 can be considered as a potential target for tumor treatment in YAP1 overexpressed breast carcinoma.

## 1 INTRODUCTION

Progressive immune dysregulation, occurring at different levels, contributes to uncontrolled tumor growth and eventually, cancer progression. A primary purpose of Cancer Immunology Research Program is to single out the proper mechanisms of anti‐tumor immunity that could instruct the development of unique and effective immunetherapies. It is well evident that the functional status of immune system has immense direct comportment on breast cancer. However, the proper mechanisms behind breast cancer pathophysiology are still not well defined.^1^ Since most of the genes in murine models, including diseases causing, are very close to human genes, it helps intensely to understand the mammalian, especially the human innate immune responses.^2^ The ability to defend against infection and destroy cancerous cells depends on lots of intracellular factors, among which the hippo signaling pathway is among the most important ones.^1^ It plays an important role in controlling cell size and number through proliferation and apoptosis.^2^ At the center of the Hippo cascade in human, is the transcription factor yes associated protein‐1 (YAP1). When activated, YAP1 translocates into the nucleus and binds transcription enhancer factors to promote the transcription of genes regulating proliferation.^2^, ^3^ Guo et al^4^ described that YAP1 suppresses cell apoptosis and encourages cell proliferation in breast cancer through the phosphatase and tensin homolog deleted 10–AKT signaling pathway. There are several reports which indicates that, YAP1 plays an important role in other types of cancers too.^5^, ^6^, ^7^

Nonetheless, the mechanisms controlling the generation and relative ratio of the functional YAP1 and other co‐factors are not well‐understood. Among those co‐factors, cytokines are the most important groups. Various literature reported that cytokines significantly influence signaling pathways to introduce epithelial immunity and regeneration, which later helps increase cancer‐related phenotypes.^8^ Cytokines belong to a group of low‐molecular‐weight polypeptide proteins and are well known for their invaluable role in inflammation and immune response regulation.^9^, ^10^ Production of abnormal cytokines and/or their receptors can result in inflammatory diseases and cancers. Some cytokines, like interleukin‐1 (IL‐1), interleukin‐6 (IL‐6) and tumor necrosis factor‐alpha (TNF‐a), are secreted during hypoxia, which is a hallmark of tumor. In inflammation and breast cancer, the role of cytokines is being investigated thoroughly.^2^, ^3^ Also, cytokines play a crucial role in the entangled link between tumor cells and tumor‐infiltrating innate and adaptive immune cells inside the tumor microenvironment.^8^, ^9^, ^11^ These tiny molecules get involved in the specific cellular functional mechanism, thus regulating signaling pathways to induce cancer.^8^, ^10^ Cytokines help to recruit immune cells to local inflammatory sites,^12^, ^13^, ^14^ resulting in the enhancement of tumor recognition by immune cells.^15^, ^16^ Cytokine molecules like IL‐1α, IL‐1β, IL6, IL‐10 inhibit and eliminate cancer^17^, ^18^, ^19^ by infiltration of immune cells in local tumor masses,^20^, ^21^ enhancing tumor immunoediting properties,^22^, ^23^, ^24^, ^25^ patronage tumor invasion and metastasis.^26^, ^27^, ^28^, ^29^, ^30^ Several reports show that cytokines take part in integrating breast cancer commencement and progression.^31^, ^32^, ^33^, ^34^ Till now, functions of cytokines and cytokine receptors have been vastly studied in murine models and to some extent in humans; a need remains for well‐thought‐out research about the contribution of cytokines in inflammation and human cancers.

Inflammation is a consequence of the defensive reaction of the body against diverse pernicious stimuli. However, the production of abnormal cytokines and their receptors can result in inflammatory diseases and cancers.^35^, ^36^, ^37^ While the profound inflammatory reaction is likely to resolve once the affront entity is decreased, this otherwise sudden and short‐term response becomes long‐lasting when the body fails to neutralize the inflammatory reactions. The inflammatory microenvironment is correlated with the secretion of various types of pro‐inflammatory and oncogenic molecules like interleukin‐1 beta (IL‐1β), interleukin‐2 (IL‐2), interleukin‐6, interleukin‐18 (IL‐18), tumor necrosis factor‐alpha (TNF‐α), interferon‐gamma (IFN‐γ), several growth factors and chemokines.^38^, ^39^ Among various cytokines, IL‐18 has emerged as a major player in inflammation and progression of different types of cancers.^40^, ^41^, ^42^, ^43^ This cytokine was first discovered as an inducing agent of IFN‐γ in the mice sera, injected with endotoxins.^2^ Different types of activated immune cells are responsible for the production of this cytokine. Among them T cells, B cells, dendritic cells, natural killer cells, macrophages and neutrophils are most important.^44^ IL‐18 induced activated T helper 1 (Th1) cells produce IFN‐γ which enhances lymphocyte proliferation, thus play a crucial role in innate and adaptive immunity regulation.^45^ Considering the host environment, IL‐18 actively takes part in the inflammatory response and immune escape of neoplastic cells.^9^, ^46^

A previous study showed that IL‐18 plays both pro and anti‐inflammatory roles in cancer progression.^9^ Yang et al^14^ reported an interesting finding which tells about the pro‐inflammatory role of IL‐18. They discovered that IL‐18 induces cell migration via down‐regulation of claudin‐12 and activation of the p38 MAPK in vitro; hence, it can be crucial in metastasis and pathogenesis in breast cancer. But on the other hand, another study showed that IL‐18 and B7‐1 molecules together increase cytolytic activity in vivo by infiltrating natural killer cells into tumors by recruiting IFN‐γ, thereby suppressing lung metastases and prolong survival.^47^ Chang et al^48^ studied recently that IL‐18 DNA, while injected intratumorally, increases the production of IFN‐γ and subsequently suppresses the liver tumor. Another study revealed that YAP1 with telomere dysfunction is involved in increased production of IL‐18 through the engagement of IFN‐γ in intestinal inflammation.^49^ They explained that telomere dysfunction of YAP1 up‐regulates pro‐IL‐18. In the gut, the microbiome‐activated caspase‐1 cleaves pro‐IL‐18 into mature IL‐18, subsequently recruiting the IFN‐γ‐secreting T cells and causes inflammation.

These findings motivated us to explore the role of YAP1 in tumor aggressiveness and immune evasion in breast invasive carcinoma and metastatic breast cancer with respect to IL‐18. We unveil an important link between IL‐18 and tumor‐derived enhanced YAP1, which leads to a gene expression profile toward tumor promotion profiling. It has also been shown the role of IL‐18 as an inducer in the production of IFNG which ultimately affects oncogenesis. Even though substantial progress has been made in treating early stage and locally advanced breast cancers, the prognosis for patients suffering from the metastatic disease remains low. Despite noteworthy advances in the treatment of malignant growth, the metastatic type of the illness remains profoundly deadly, with a 5‐year generally endurance pace of just around 20%.^50^

About 70% of patients with metastatic sicknesses ineluctably become impervious to treatment.^51^ Advances in disease immunotherapy with resistant checkpoint blockers have indicated that bridling the power of the body's invulnerable framework can be a useful technique to battle metastatic malignancy.

## 2 MATERIALS AND METHODS

For our study, we used the data generated by The Cancer Genome Atlas (TCGA) Research Network: http://cancergenome.nih.gov/. The TCGA program has created a profuse amount of data that will help to discover the DNA copy number alterations, mRNA regulation levels, DNA methylation alteration, and somatic mutations in DNA among the patients of 20 different types of cancers.^52^ Though the numbers and types of data accompanied by other clinical variables sometimes differ with the particular group of tumor tissues, TCGA with huge data sources has been proven as a potential platform for the study and assessment of genetic changes. We selected two datasets; Breast Invasive Carcinoma (TCGA, PanCancer Atlas, *n* = 1084 patients) and Metastatic Breast Cancer (INSERM, PLoS Med 2016, *n* = 216 patients) for our study.^53^ The sample types are tumors from the biopsy. We queried three genes *IL‐18*, *YAP1* and *IFN‐γ*, for the indicated cancer study. The overall survival was obtained following the Kaplan–Meier method and comparison between curves were made using Log‐Rank test. The *p* values were determined by Fisher's exact test with the null hypothesis. Correlation plots illustrating the relationship between mRNA expression levels in the sample were analyzed by comparison with gene copy numbers from the GISTIC2.0 (Genomic Identification of Significant Targets in Cancer) available through cBioportal.^54^

All the molecular analyses were performed by using the web‐based tools through cBioportal website (http://www.cbioportal.org/).

## 3 RESULTS

**FIGURE 1:**
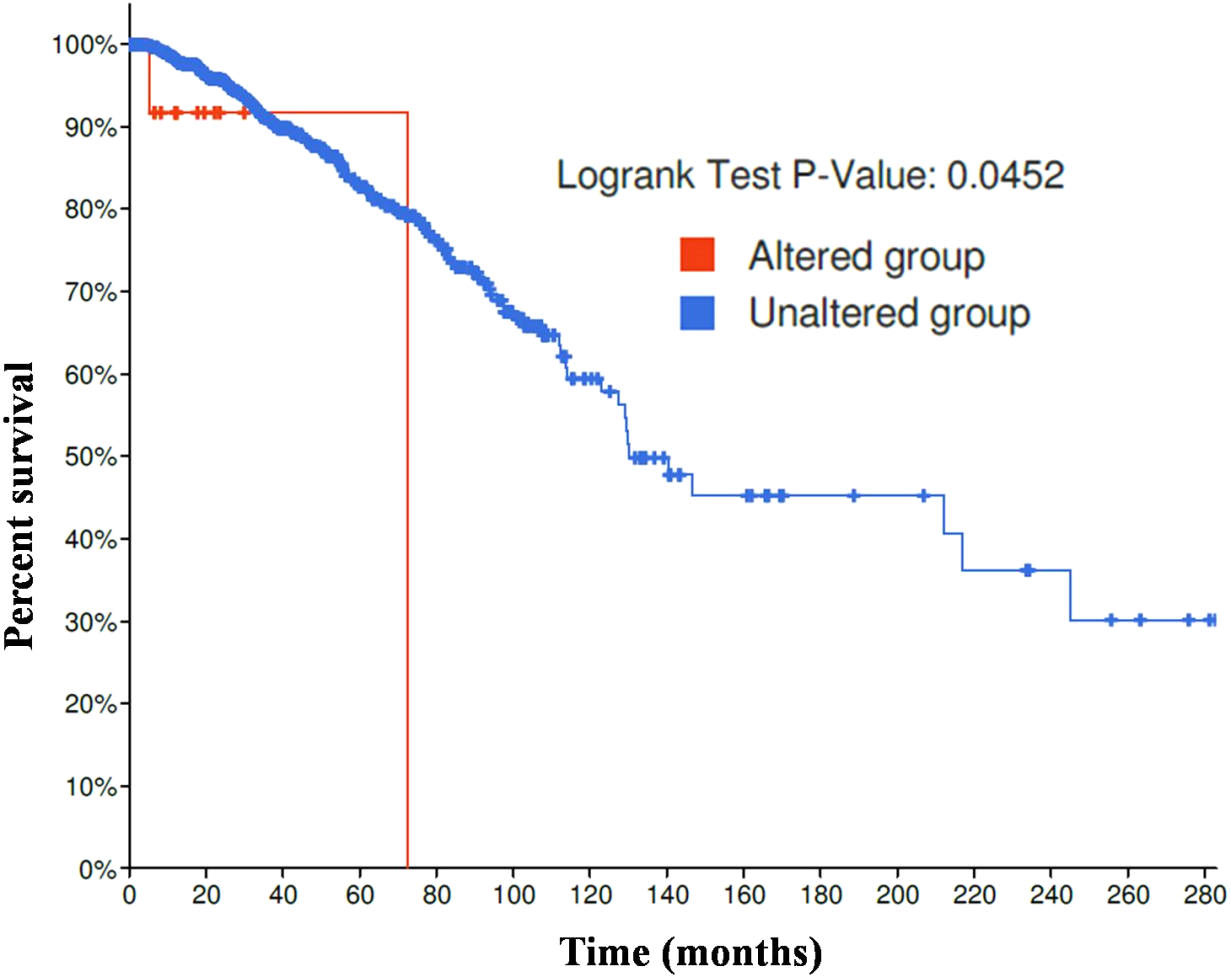
IL18 mutation effects on overall survival in Breast Invasive Carcinoma (TCGA, PanCancer Atlas) cohort. Logrank test was performed for overall survival analysis demonstration. Red line indicates survival in patients with *IL‐18* mutation, and the Blue line represents patients' survival in with no mutations in the given gene

Molecular alterations of the tumor cells result in the expression of tumor markers and can be used to the immune system to locate new associations for a better prognosis. To evaluate whether altered expression of the *IL‐18* gene have an effect on *YAP1*, associated with breast cancer progression and metastasis, we studied the expression of *IL‐18*, *IFNG* and *YAP1* genes. We found that overall survival is better in unaltered *IL‐18* group patients than altered group (Figure 1).

**FIGURE 2:**
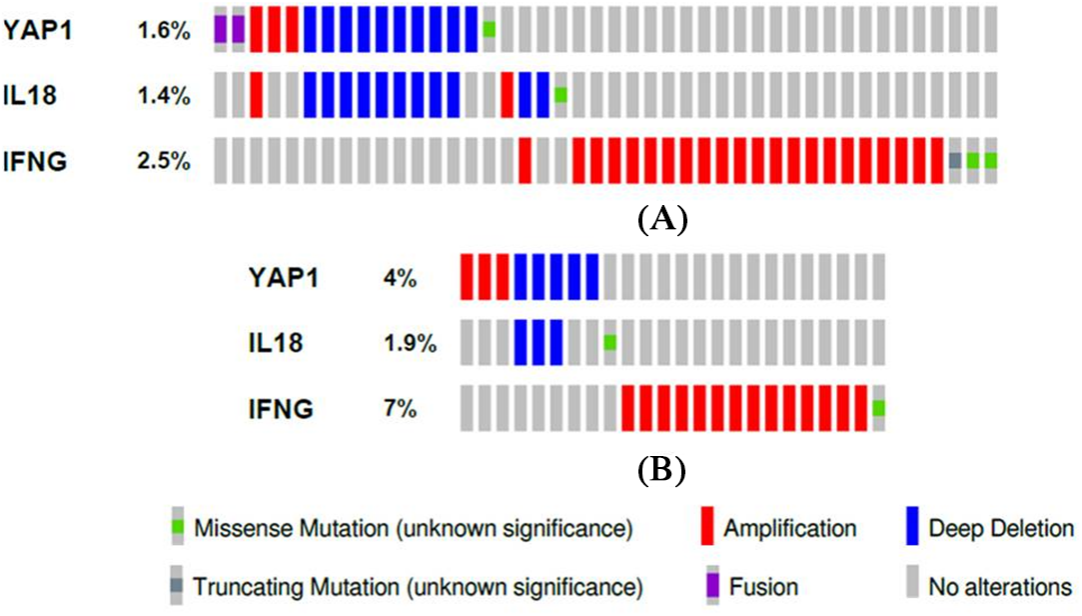
Oncoprint represents the changes in gene expression level in Breast Invasive Carcinoma (TCGA, PanCancer Atlas) samples for the three genes (*YAP1*, *IL‐18* and *IFNG*) involved. Expression of genes analysis was done by using cBioportal. (A) cBio Oncoprint of patients of Breast Invasive Carcinoma cohort (TCGA, PanCancer Atlas; *n* = 996 patients) (http://www.cbioportal.org/) and (B) Metastatic Breast Cancer cohort (INSERM, PLoS Med 2016) (*n* = 216 patients). Alteration types and levels against each gene are represented in each horizontal row. Individual patient sample data is oriented vertically. Samples without alterations were eliminated from the oncoprint figure

We queried two cohorts, Breast Invasive Carcinoma (TCGA, PanCancer Atlas, *n* = 1084 patients) and Metastatic Breast Carcinoma (INSERM, PLos Med 2016, *n* = 216 patients) and found evidence of *YAP1* and *IL‐18* gene alteration (Figure 2A) 1.6 and 1.4% respectively among the patients of Breast Invasive Carcinoma (TCGA, PanCancer Atlas) and 4% and 1.9% (Figure 2B) respectively in Breast Metastatic Carcinoma (INSERM, PLos Med, 2016) cohort. As expected, *IL‐18* induced *IFNG* gene alteration has been increased dramatically in metastatic breast carcinoma (Figure 2B), which denotes the failure of *IL‐18* to stop genetic alteration of *IFNG* in advanced breast cancer. Whereas in primary breast cancer, the genetic alteration of *IFNG* is significantly low and *IL‐18* and *YAP1* showed almost same level of gene alteration (Figure 2A).

Further, again we performed Oncoprint analysis using cBioportal (http://www.cbioportal.org/), (https://github.com/cBioPortal/cbioportal/blob/master/docs/News.md)^55^, ^56^ to find out the expression patterns and any possible genetic alteration for the foretasted immune modulatory molecules in breast cancer patient tumors. One can get a precise graphical summary of genetic alteration of different types of genes over a particular group of tumor samples by performing oncoprint analysis.^57^ We conducted oncoprint analysis through cBioportal's oncoprint tool within 996 primary breast tumor samples among three desired genes to compare the differences in expression magnitude (Table 1). This table shows both numbers as well as the types of gene alteration along with the tendency of occurrence in the same patient. Although, when performed overall survival analysis in single or three genes, none of them showed a significant correlation (Logrank test *p*‐value = .0855) or disease‐free survival (Logrank test *p*‐value = .820; data not shown). We found 0.3% amplification, 1% deep deletion of *YAP1* in Breast Invasive Carcinoma (TCGA, PanCancer Atlas cohort) especially, breast invasive mixed mucinious carcinoma subtype. In this cohort, the most amplified gene is *IFNG*, which is 2.21% and mostly abandoned in breast invasive ductal carcinoma. Whereas, *IL‐18* showed the highest deep deletion, about 7.69% in breast invasive mixed mucinious carcinoma. *YAP1* showed 0.3%, *IL‐18* showed 0.1% and *IFNG* showed 0.3% somatic mutation (data not shown). On the other hand, we detected 1.39% amplification and 2.31 deep deletions in *YAP1* and 0% somatic mutation in Metastatic Breast Cancer cohort (INSERM, PLoS Med 2016). In same cohort, *IL‐18* showed 1.39% deep deletion and 0.5% somatic mutation. An exhibition of inclination toward mutual exclusivity has been observed among the altered genes in the foretasted cohorts. Table 2 summarized the statistical data on mutual exclusivity and co‐occurrence in each pair of queried altered gene in Breast Invasive Carcinoma (TCGA, PanCancer Atlas). The strongest co‐occurrence nature has been observed in *YAP1‐IL‐18* pair with a statistically significant value *p* < .001. On the other hand, *IL‐18‐IFNG* and *YAP1‐IFNG* gene pairs showed no mutual exclusivity (*p* = .301 and *p =* .664 respectively). In this study, the Fisher's exact test along with null hypothesis calculated all the *p* values. In metastatic breast cancer (Table 3), *YAP1‐IL‐18* showed the strongest co‐occurrence (*p* < .001); on the other hand, *IL‐18‐IFNG* and *YAP1‐IFNG* showed insignificant mutual exclusivity of genomic alterations (*p* = .748 and *p =* .557, respectively).

**TABLE 1:** Summary of mutational and expression data of three genes from breast invasive carcinoma (TCGA, PanCancer Atlas) cohort

	YAP1	IL18	IFNG
Total changes (%)	1.6	1.4	2.5
mRNA up (%)	3.62	2.82	3.22
mRNA down (%)	2.72	0	0
Fusion (%)	0.2	0	0
Copy increase (%)	0.3	0.2	2.21
Deep deletion (%)	1.0	1.1	0

*Note*: Information about the number and alteration types of three genes taken for study.

**TABLE 2:** The alterations in three genes are distributed in a nearly mutually exclusive way across samples in Breast Invasive Carcinoma (PanCancer Atlas) cohort

A	B	Neither	A not B	B not A	Both	Log2 odds ratio	*p* Value	*q* Value	Tendency
YAP1	IL18	976	6	4	10	>3	<.001	<0.001	Co‐occurrence
IL18	IFNG	958	13	24	1	1.618	.301	0.452	Co‐occurrence
YAP1	IFNG	955	16	25	0	<−3	.664	0.664	Mutual exclusivity

*Note*: Only, the *YAP1‐IL‐18* pair has the strongest tendency toward co‐occurrence and it is statistically significant (*p* < .001). For the other two gene pairs *IL‐18‐IFNG* and *YAP1‐IFNG* it is not statistically significant (*p* = .301 and *p =* .664, respectively).

**TABLE 3:** Alteration distributions among three genes are nearly in a mutually exclusive way across the samples. It has been analyzed statistically and visualized with the Mutual Exclusivity tool via cBioportal in Metastatic Breast Cancer (INSERM PLos Med, 2016) cohort

A	B	Neither	A not B	B not A	Both	Log2 odds ratio	*p* Value	*q* Value	Tendency
YAP1	IL18	207	5	1	3	>3	<.001	<0.001	Co‐occurrence
IL18	IFNG	197	4	15	0	<−3	.748	0.748	Mutual exclusivity
YAP1	IFNG	193	8	15	0	<−3	.557	0.748	Mutual exclusivity

*Note*: *YAP1‐IL‐18* showed the strongest co‐occurrence (*p* < .001); on the other hand, *IL‐18‐IFNG* and *YAP1‐IFNG* showed insignificant mutual exclusivity of genomic alterations (*p* = .748 and *p =* .557, respectively).

**FIGURE 3:**
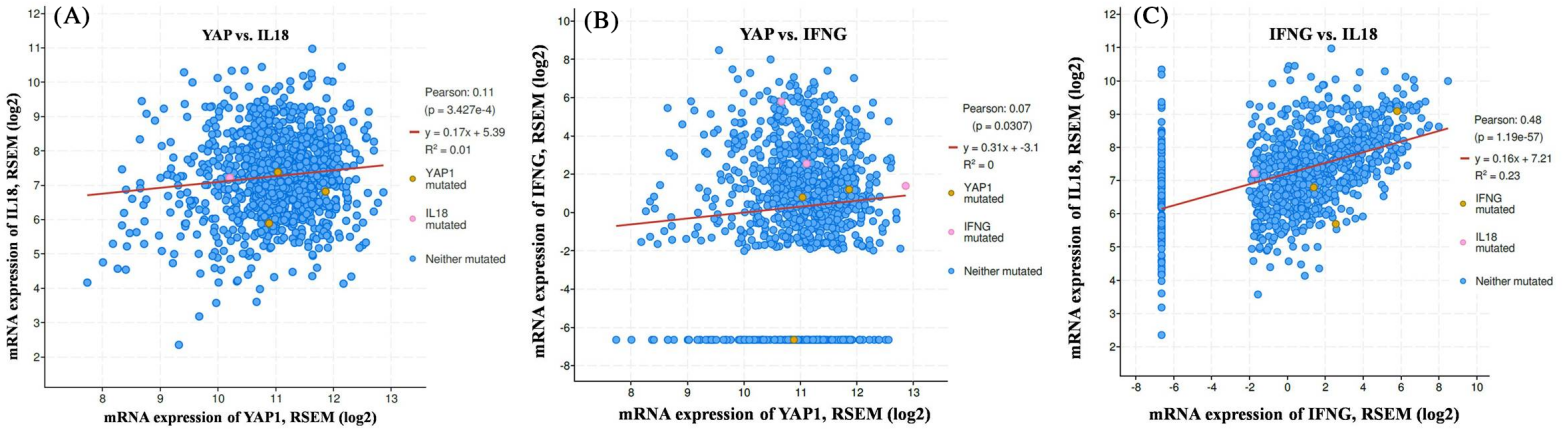
Correlation plots for co‐occurrence and correlation of mRNA for (A) YAP1 versus IL‐18 (B) *YAP*1 versus IFNG and (C) IFNG versus IL‐18

To get the gene expression pattern correlated with *YAP1* across the patients in Breast Invasive Carcinoma (TCGA, PanCancer Atlas) cohort, we used cBioprtal Co‐expression tool. We found two genes, *IL‐18* and *IFNG* are correlated with *YAP1* and the expression of *YAP1* and *IL‐18* was positively correlated (Figure 3A; Pearson: *P* = .11, *p* = 3.427e‐4), but there is no correlation with *YAP1* and *IFNG* (Figure 3B; Pearson: *P* = .07, *p* = .0307). Figure 3C showed that the gene pair IL18 and IFNG are strongly correlated (Pearson: *P* = 0.48, p = 1.19e‐57).

**FIGURE 4:**
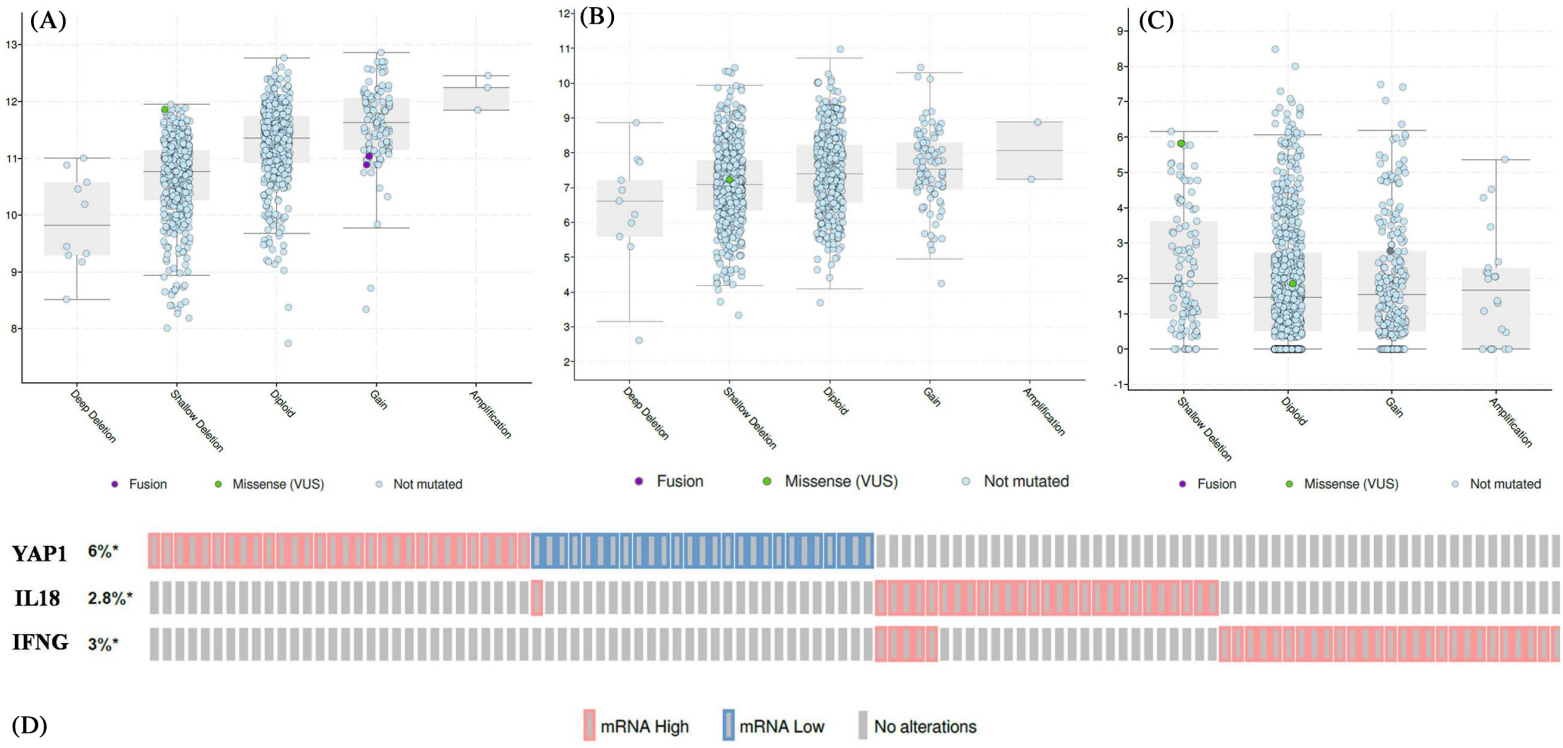
Distribution of genomic alterations in breast tumors involve homozygous deletions (deep loss), heterozygous deletion (shallow loss), and normal diploid and low‐level gain (amplification). Correlation plots illustrating the relationship between mRNA expression levels and gene copy number for (A) YAP1, (B) IL‐18 and (C) IFNG. The putative copy number changes displays on *X*‐axes and the *Y*‐axes (log base 2) represents the mRNA expression level. Possible scores of GISTIC2 obtained from cBioportal are likely; 2 = deep (homozygous) deletion; −1 = shallow (hemizygous) deletion; 0 = neutral/diploid; 1 = gain (low copy); 2 = high level amplification. (D) Oncoprint displays mRNA expression level for the three genes involved in breast invasive carcinoma. The “*” represents significant value

Identification of putative copy‐number alterations from GISTIC^54^ has shown that in YAP1, there was a trend toward deep deletions versus gains (Figure 4A). The same trend has been noticed for IL‐18, too (Figure 4B). But in IFNG, there is a trend toward shallow deletion versus gains (Figure 4C). Most of these breast cancer tissues exhibited increased DNA copy numbers, reflecting its consistent increased mRNA expression. In addition, the overall oncoprint of *YAP1*, *IL‐18* and *IFNG* gene expressions in Breast Invasive Carcinoma (PanCancer Atlas) cohort showed that mRNA expression was up‐regulated by 3.02, 2.82, and 3.22% (YAP1, IL18 and IFNG) in all patients, respectively (Figure 4D). Almost half of the parts of the population (2.72%) have been seen YAP1 down regulation. Therefore, our data support that *YAP1* was increased in breast cancer (Figure 2B), which might mainly originate from its increased DNA copy number and almost unchanged IL‐18.

## 4 DISCUSSION

Quite many investigations have detailed that IL‐18 expression level is positively correlated with disease progression in different types of cancers.^42^, ^58^, ^59^ IL‐18 is a cytokine that manipulates both pro and anti‐cancer characteristics depending upon the host condition.^9^, ^45^ Scientific studies say that in IL‐18 overexpressed murine model with malignant melanoma, it reduces the rate of tumor development, enhances apoptosis in tumor cells and lessens lung metastasis. This study suggests that IL‐18 possesses an anti‐cancer property.^60^ A recent study showed that IL‐18 pathway could be implicated for immunotherapeutic intervention to enhance antitumor immunity.^61^, ^62^ The outcome of our analysis suggests that IL‐18 probably has anti‐cancer property on YAP1 overexpressed tumor cells which can be further clarified by in vitro studies. Our results suggest that a better prognosis in breast cancer is proportionate to higher expression of IL‐18 (Figure 1). It also suggests an inferior prognosis in breast cancer patients eventually because of higher expression of YAP1 and lower level of IL‐18 (Figure 1 and Figure 2A,B). Our finding also exhibited a positive correlation between IL‐18 and YAP1 via IFNG gene expression and showed that IL‐18 expression is positively correlated with YAP1 in breast invasive carcinoma (Figure 3A). We identified putative copy‐number alterations from GISTIC, which showed the most common events reported in Breast Invasive Carcinoma (TCGA, PanCancer Atlas), is the gain of copy number followed by heterozygous deletion (Figure 4A–C). Figure 2B showed an increased YAP1 DNA copy number and almost unchanged IL18 DNA copy number results in metastasis.

## 5 CONCLUSION

In conclusion, the present data analysis suggests that IL‐18 might influence YAP1 in Breast oncogenesis through the production of IFNG and IL‐18 expression is positively correlated with YAP1 expression in breast invasive carcinoma. Thus, based on our analysis, we hypothesized that IL‐18 either acts as a regulatory factor in the expression of YAP1 or interacts with it to determine the occurrence and progression in breast carcinoma. Taken together, the present data provide crucial clinically significant information that IL‐18 expression might become an important prognostic feature in YAP1 overexpressed breast invasive carcinoma and can be considered as a potential target for tumor treatment. Further, it will be interesting to perform in vitro as well as in vivo studies to explore the possible link between YAP1 and IL‐18 for a better understanding of cancer progression.

## CONFLICT OF INTEREST

The authors declare there is no conflict of interest.

## AUTHOR CONTRIBUTIONS

A.R., and L.S.S. conceived and designed the study. A.R. acquired the data. A.R. performed the analysis and interpreted the data. A.R. and L.S.S. wrote the article. All the authors reviewed the article and gave final approval of the version to submit for publication.

## ETHICAL STATEMENT

Not applicable.

## Data Availability

Details of the data and analysis used in this study are available on reasonable request from the corresponding author.
